# Multimorbidity and risk of adverse outcomes in the Hertfordshire Cohort Study: does sex matter?

**DOI:** 10.1007/s40520-024-02874-9

**Published:** 2024-11-14

**Authors:** Leo D. Westbury, Roshan Rambukwella, Camille Pearse, Kate A. Ward, Cyrus Cooper, Elaine M. Dennison

**Affiliations:** 1 MRC Lifecourse Epidemiology Centre, University of Southampton, Southampton, UK; 2 NIHR Southampton Biomedical Research Centre, University of Southampton and University Hospital Southampton NHS Foundation Trust, Southampton, UK; 3 NIHR Oxford Biomedical Research Centre, University of Oxford, Oxford, UK; 4 Victoria University of Wellington, Wellington, New Zealand

**Keywords:** Epidemiology, Comorbidity, Hospital admission, Mortality

## Abstract

**Aims:**

We examined whether admission risk increases at a certain threshold of number of systems medicated or whether any increase confers greater admission risk in either sex in a community-dwelling cohort of older persons in Hertfordshire. This study uses a longitudinal retrospective study design.

**Methods:**

Data from 2997 men and women (aged 59–73 at baseline) were analyzed. Participants were followed up from baseline (1998–2004) until December 2018 using Hospital Episode Statistics and mortality data, reporting clinical outcomes using ICD-10 coding. Number of systems medicated in relation to mortality (all-cause, cancer-related, cardiovascular-related) and admission (any, neurological, cardiovascular, and respiratory) were examined using Cox regression.

**Results:**

Apart from cancer-related mortality among women, more systems medicated predicted increased risk of all events among both sexes (*p* ≤ 0.001). For ‘any’, cardiovascular and respiratory admissions, there were increases in risk for each category of number of systems medicated. For example, compared to men with no systems medicated, those with 1, 2 and > 2 systems medicated had hazard ratios (95% CI) for cardiovascular admissions of 1.82 (1.57,2.12), 2.39 (2.00,2.84) and 3.45 (2.84,4.20) respectively; estimates among women were 1.74 (1.44,2.11), 2.35 (1.92,2.88) and 3.40 (2.79,4.13).

**Conclusions:**

Increases in numbers of systems medicated conferred greater risk of admission in both sexes. Interventions aimed at reducing the burden of chronic disease in mid-late adulthood are required.

**Supplementary Information:**

The online version contains supplementary material available at 10.1007/s40520-024-02874-9.

## Background

The demographic shift towards more people living to older ages brings numerous opportunities and challenges; one of the most pressing issues for planners of healthcare provision is the increased prevalence of multimorbidity among older persons [1]. Multimorbidity refers to the coexistence of two or more chronic disease conditions within an individual [2]. Prevalence estimates for multimorbidity range from 20 to 30% in “all age” populations and may be as high as 55–98% in older populations [3]. Prevalence estimates are expected to rise further due to ageing populations, and improvements in medical technology [3].

In previous studies, people with multimorbidity have been shown to have poorer health outcomes, including increased hospital admissions [4–8]. The total expenditure on healthcare in high-income countries is dominated by the needs of those with multiple long-term conditions [6, 9, 10]. While multimorbidity is not restricted to older adults, with some studies even finding higher absolute numbers of individuals with multimorbidity in younger age groups [11], it is more prevalent among older people [7]. Therefore, the healthcare demands and costs associated with multimorbidity will continue to rise as populations age [11].

Hence addressing multimorbidity during midlife may reduce the burden on healthcare systems and improve the overall quality of life for individuals as they age [12, 13]. Furthermore, understanding relationships between multimorbidity and hospital admissions might facilitate a research agenda into the benefits of interventions at a personal and societal level.

Despite this issue being recognised as important to public health and resource utilisation, to our knowledge, no previous study has considered sexual dimorphism in the relationship between multimorbidity and risk of subsequent hospital admission. We hypothesised that these may be present, as the profile of common comorbidities is different in men and women, with cardiovascular diseases such as coronary heart disease being more common in men [14], while for example musculoskeletal diseases such as osteoporosis and osteoarthritis are more common in women [15, 16]. Furthermore, health seeking behaviours are different in the two sexes, with women reported as higher users of health services [17].

## Aims

Hence although multimorbidity is well established as an important contributor to risk of hospital admission, it is unclear whether risk of adverse health events only increase once a certain level or threshold of comorbidity burden is reached, or whether any increase in comorbidity level, confer greater risks of these events, and whether such relationships vary by sex. Such information would be helpful when planning future research and possible interventions. We considered this in this study that relates number of systems medicated (a marker of comorbidity level) to risk of mortality and hospital admission events over 20 years of follow up among participants from the Hertfordshire Cohort Study (HCS), a population of community-dwelling older people.

## Methods

### The Hertfordshire Cohort Study

The Hertfordshire Cohort Study (HCS) consists of 2997 individuals who were born in Hertfordshire (UK) in the 1930s. They attended a clinic visit and home interview in 1998–2004 for a detailed health assessment. The HCS had ethical approval from the Hertfordshire and Bedfordshire Local Research Ethics Committee; all participants provided informed consent for the investigations they underwent and for researchers to access their medical records in the future. Additional details about this study have been previously published [18].

### Ascertainment of participant information at baseline (1998–2004)

A nurse-administered questionnaire was used to obtain information on alcohol consumption and smoking. Occupational social class was ascertained from most recent or current full-time occupation for men and among women who never married, and from husband’s occupation for ever-married women. Occupations were then classified according to the 1990 OPCS Standard Occupational Classification (SOC90) unit group for occupation [19]. Details of all over-the-counter and prescription medications currently taken were recorded; these were coded according to the British National Formulary into the following systems: cardiovascular; respiratory; gastro-intestinal; endocrine; central nervous; malignant disease and immunosuppression; nutrition and blood; musculoskeletal and joint disease; eye; ear; nose; skin; miscellaneous; and genito-urinary tract. The number of systems each participant was taking medications for was then derived; this was used as a marker of morbidity level as has been done previously in this cohort [20–22]. Measures of morbidity burden based on medications taken are widely used and are strongly associated with adverse health outcomes [23].

At the baseline clinic, participants had their height (Harpenden pocket stadiometer, Chasmors Ltd, London, UK) and weight (SECA floor scale, Chasmors Ltd, London, UK) measured; these measurements were used to derive BMI.

### Ascertainment of adverse health events

These events were identified using Hospital Episode Statistics (HES) data and mortality data. The Ethics and Confidentiality Committee of the National Information Governance Board and NHS Digital granted permission to obtain these data from HCS participants from 01/04/1998 to 31/12/2018. Underlying causes of death were identified using ICD-10 codes and defined as follows: Cancer (C00-C97); Cardiovascular (I10-I79); and Other (other ICD-10 codes). The linkage of the HCS cohort with HES data has been described previously [24]; the HES data extract for each participant included information relating to their hospital admissions such as the admission date, diagnoses coded to ICD-10, and date of discharge. Admission types were identified by ICD-10 codes assigned to each admission and were defined as follows: Neurological – G00-G99 (diseases of the nervous system); Cardiovascular – I10-I79; and Respiratory – J00-J99 (diseases of the respiratory system).

### Statistical methods

Descriptive statistics were used to describe key baseline participant characteristics and the health events experienced during follow-up. Number of systems medicated in relation to risk of adverse events relating to mortality (all-cause, cancer-related, cardiovascular-related, and mortality not due to cancer or cardiovascular causes) and hospital admission (any, neurological, cardiovascular and respiratory) were examined using time-to-first event Cox regression. The proportional hazards assumption for the Cox models was confirmed by plotting the scaled Schoenfeld residuals against time. Competing risk regression for the hospital admission events as a sensitivity analysis was performed using the Fine-Gray subdistribution hazards model with death as a competing risk [25]. For all survival analyses, follow-up time started from the HCS baseline clinic (ranging from 1998 to 2004, depending on each participant) and ended on 31st December 2018. Analyses were conducted using Stata, release 17.0; *p* < 0.05 was regarded as statistically significant. All analyses were stratified by sex. If an individual had missing values for variables required for the survival analysis of a particular outcome, for example, the follow-up time variable, then they were not included in the analysis of that particular outcome. However, the proportion of missing values for each adverse health outcome was very low and ranged from 0 to 0.13%.

## Results

### Descriptive statistics

Table 1 presents descriptive statistics for the baseline participant characteristics and adverse health events during follow-up. Mean (SD) age at baseline was 65.7 (2.9) among men and 66.6 (2.7) among women. Overall, 32% of men and 24% of women had no systems medicated at baseline whereas 31% of men and 44% of women had two or more systems medicated. During follow-up, 93% of men and 92% of women had at least one hospital admission; 36% of men and 26% of women died during follow-up.

**Table 1:** Baseline participant characteristics and adverse health events during follow-up

Participant characteristic [mean (SD), median (lower quartile, upper quartile), or %]	Men (*n* = 1579)	Women (*n* = 1418)
***Characteristics at baseline (1998–2004)***		
Age (years)	65.7 (2.9)	66.6 (2.7)
BMI (kg/m^2^)	27.2 (3.8)	27.6 (4.9)
Ever smoked regularly	67%	39%
High alcohol intake (units per week: ≥22 men, ≥ 15 women)	22%	5%
Social class (manual)	59%	58%
Number of systems medicated		
0	32%	24%
1	37%	32%
2	19%	21%
3 or more	12%	23%
**Events during follow-up (ever had)**		
Death (all-cause)	36%	26%
Death (cancer-related)	15%	11%
Death (cardiovascular-related)	11%	5%
Death (other)	11%	10%
Hospital admission	93%	92%
**Types of admission during follow-up (ever had)**		
Neurological	23%	20%
Cardiovascular	71%	68%
Respiratory	40%	34%

Follow-up period lasted from baseline (1998–2004) until 31st December 2018

### Number of systems medicated in relation to adverse health events

Hazard ratios (95% CI) for adverse health events per additional system medicated at baseline are presented in Table 2. For some events, such as all-cause mortality, cardiovascular admissions and respiratory admissions, greater hazard ratios were observed among men compared to women. The relationship between number of systems medicated and risk of cancer-related mortality among women was weak (*p* = 0.574). However, for all other events, a significant increase in risk was observed for higher numbers of systems medicated (*p* ≤ 0.001); this was the case among men and women. For example, the hazard ratio (95% CI) for a cardiovascular admission per additional system medicated was 1.40 (1.34, 1.47) among men and 1.33 (1.28, 1.39) among women.

**Table 2:** Hazard ratios (95% CI) for adverse health events per additional system medicated at baseline

Health event	Men		Women	
	Hazard ratio (95% CI)	*P*-value	Hazard ratio (95% CI)	*P*-value
Death (all-cause)	1.37 (1.29,1.46)	< 0.001	1.18 (1.11,1.26)	< 0.001
Death (cancer)	1.19 (1.07,1.33)	0.001	1.03 (0.92,1.15)	0.574
Death (cardiovascular)	1.55 (1.39,1.73)	< 0.001	1.34 (1.17,1.55)	< 0.001
Death (other)	1.44 (1.28,1.61)	< 0.001	1.26 (1.14,1.40)	< 0.001
Admission (any)	1.23 (1.18,1.29)	< 0.001	1.23 (1.19,1.28)	< 0.001
Admission (neurological)	1.32 (1.22,1.44)	< 0.001	1.23 (1.15,1.33)	< 0.001
Admission (cardiovascular)	1.40 (1.34,1.47)	< 0.001	1.33 (1.28,1.39)	< 0.001
Admission (respiratory)	1.46 (1.38,1.55)	< 0.001	1.30 (1.24,1.38)	< 0.001

Hazard ratios were derived from time-to-first event Cox regression models

Death was regarded as a censoring event for hospital admission events

Other causes of death were those that were not from cancer or cardiovascular causes

**Fig. 1:**
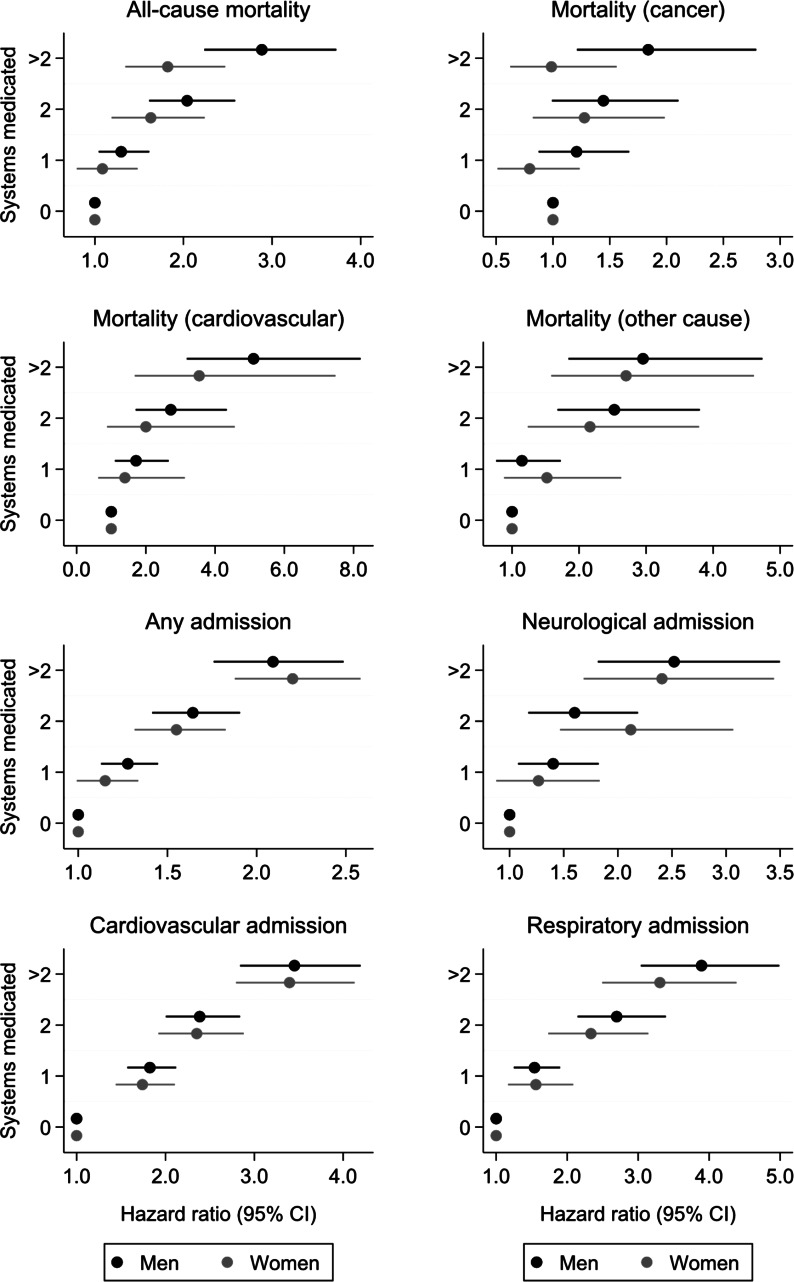
Hazard ratios (95% CI) for adverse health events according to number of systems medicated at baseline (reference category of no systems medicated). Time-to-first event Cox regression was used; death was regarded as a censoring event for hospital admission outcomes. Other causes of death were those that were not from cancer or cardiovascular causes

Figure 1 presents hazard ratios (95% CI) for adverse health events according to number of systems medicated at baseline compared to a reference category of no systems medicated. For several types of hospital admissions (any, cardiovascular admissions, and respiratory admissions), there were clear increases in risk for each higher category of number of systems medicated (0, 1, 2, > 2) with similar effect estimates among men and women. For these outcomes, there were no threshold effects where the risk of the event only increased when a certain number of systems medicated was reached. For example, compared to men with no systems medicated, those with 1, 2 and > 2 systems medicated had hazard ratios (95% CI) for cardiovascular admissions of 1.82 (1.57, 2.12), 2.39 (2.00, 2.84) and 3.45 (2.84, 4.20) respectively; corresponding estimates among women were 1.74 (1.44, 2.11), 2.35 (1.92, 2.88) and 3.40 (2.79, 4.13).

### Sensitivity analyses

Supplementary Table 1 shows associations between number of systems medicated and risk of hospital admission events which have been examined using competing risk regression with death as a competing risk. These associations were similar to those estimated using time-to-first event Cox regression models.

## Discussion

Our results clearly link multimorbidity, measured using number of systems medicated, with risk of mortality and hospital admission events. Except for carcinoma-related deaths, we observed that greater numbers of systems medicated was strongly associated with increased risk of all other outcomes relating to mortality and hospital admissions among both men and women. Specifically, for key hospital admission events (any admissions, cardiovascular admissions and respiratory admissions), there were no threshold effects where the risk of the event only increased when a certain number of systems medicated was reached; instead, increases in number of systems medicated, even from a low base, conferred greater risks of these events. Patterns of association were again similar among men and women. More systems medicated was not associated with increased risk of cancer-related deaths among women. This could be due to the fairly small proportion of women who experienced this outcome.

Our findings in this study accord with, and extend, previous work. A previous retrospective cohort study of 180,815 patients, aged 20 years and older based in Scotland, found that unplanned and potentially preventable unplanned admissions were independently associated with increasing physical multimorbidity [26] while another study that reported associations of multimorbidity and income with hospital admission in three widely differing health care systems (Scotland, China, and Hong Kong) found that multimorbidity increased odds of admissions in all three settings [13]. Reed and colleagues performed a root cause analysis of causes of unplanned hospital admissions of patients with multimorbidity in Australia, reporting that there were six main causes; a consequence of minimal care, progression of disease, home care accessibility, high complexity, clinical error, and delayed care-seeking by the patient [27]. Public health interventions to reduce adverse clinical outcomes in patients with more than one comorbidity might consider how we can address these factors in the community.

We studied relationships between multimorbidity in late midlife and events over 20 years of follow-up; hence our findings add to the literature that has considered other stages of the lifecourse. Lai and colleagues examined routine clinical records of all patients aged 45 + years with chronic conditions discharged from public general hospitals in Hong Kong. They studied patterns of annual frequencies of hospital admissions and number of hospitalized days over nine years and compared these according to multimorbidity status and age group. Interestingly they found that on interaction analysis, the effect of multimorbidity on hospitalization was stronger in younger groups, highlighting the need to consider the consequences of multimorbidity throughout the lifecourse [12]. Work from the Newcastle 85 + Study found that morbidity load was related to medication burden and use of some, but not all, healthcare services [3]. Although it has been suggested that identifying participant clusters sharing similar morbidity profiles might help inform future healthcare provision and the identification of common underlying biological mechanisms [3], Stokes et al. [10] failed to identify clear “high cost” combinations of multimorbidity as possible targets for intervention so further work is needed in this area. We recognise that a limitation of our study was an inability to consider this aspect further.

Our findings highlight the importance of addressing risk factors for chronic diseases in midlife to prevent adverse health events in later life. Since common chronic diseases, including cardiovascular, respiratory and malignant conditions, and diseases such as diabetes share common lifestyle risk factors including smoking, lack of physical activity, high alcohol consumption and poor diet quality, public health measures can be targeted to address them especially at primary care level [28, 29]. This has been proven to be strategically effective in reducing adverse health events requiring hospitalization [30].

Our study has several strengths and limitations. As we used data that were routinely collected by the HES service for England, we had information on admissions in NHS hospitals and NHS care in private hospitals. A limitation of this approach is that we do not have any information on private health care. This approach did mean that we have complete follow up of our cohort, with the limitation previously noted. The data available relate only to inpatient admissions and we have no information on outpatient care where no admission was required. Some selection bias may have occurred as our study was conducted in a single county (Hertfordshire) with fairly low levels of deprivation and participants were all White Caucasian so findings may be less generalizable to community-dwelling older people of other ethnic groups or living elsewhere. However, characteristics of HCS participants have previously been found to be broadly comparable to those in the Health Survey for England [31]. Another limitation is that few individual comorbidities were ascertained at the baseline stage of HCS. Therefore, we used number of systems medicated as a marker of morbidity level. Although, measures of morbidity burden based on medications have been used in previous studies and are related to risk of adverse outcomes [23], these measures may be a poorer proxy of disease burden when defined at the system-level, rather than at the level of individual conditions, and may not capture conditions which are managed by non-pharmacological means. Furthermore, our study had a limited ability to examine associations between specific clusters of comorbidities and outcomes, as has been undertaken in one other study [9]. Finally, relationships reported may have been affected by residual confounding or confounding by indication as participants with many systems medicated may differ in other ways between those without, aside from simply having higher levels of comorbidity.

## Conclusions

Our study sheds light on the issue of multimorbidity in older people and its impact on hospital admissions. It builds on previous work by allowing us to consider whether a threshold of number of systems medicated confers much higher risk for adverse clinical events if breached. Instead we observed a graded relationship between number of systems medicated and risk of admission in both men and women, highlighting the need to implement public health measures earlier in life to reduce comorbidity level in mid-late adulthood. These could include interventions that promote physical activity to reduce obesity and risk of diabetes and cardiovascular disease, as there does not appear to be a ‘safe’ level of comorbidity.

## Electronic Supplementary Material

Below is the link to the electronic supplementary material.

Supplementary Material 1

## Data Availability

Data relating to this study cannot be shared due to consent restrictions.
