# Supplementary material for: Multimorbidity and risk of adverse outcomes in the Hertfordshire Cohort Study: does sex matter?
Source: Aging Clin Exp Res. 2024 Nov 14;36(1):218. doi: 10.1007/s40520-024-02874-9 (PMC11564394; doi:10.1007/s40520-024-02874-9)
Supplement: Supplementary file 1 — Supplementary Material 1 [file 40520_2024_2874_MOESM1_ESM.docx]

**Online Resource**

**Article title:** Multimorbidity and risk of adverse outcomes in the Hertfordshire Cohort Study: does sex matter?

**Journal:** Aging Clinical and Experimental Research

**Authors**: LD Westbury, R Rambukwella, C Pearse, KA Ward, C Cooper, EM Dennison

**Main affiliation and e-mail address for corresponding author (Elaine Dennison)**:

MRC Lifecourse Epidemiology Centre, University of Southampton, Southampton, UK

[emd@mrc.soton.ac.uk](mailto:emd@mrc.soton.ac.uk)

| **Supplementary Table 1: Subhazard ratios (95% CI) for adverse health events per additional system medicated at baseline** | | | | |
| --- | --- | --- | --- | --- |
|  |  |  |  |  |
|  |  |  |  |  |
| **Health event** | **Men** | | **Women** | |
|  | **Subhazard ratio (95% CI)** | **P-value** | **Subhazard ratio (95% CI)** | **P-value** |
| Admission (any) | 1.22 (1.16,1.28) | <0.001 | 1.21 (1.15,1.28) | <0.001 |
| Admission (neurological) | 1.21 (1.11,1.32) | <0.001 | 1.20 (1.12,1.29) | <0.001 |
| Admission (cardiovascular) | 1.35 (1.28,1.43) | <0.001 | 1.30 (1.23,1.37) | <0.001 |
| Admission (respiratory) | 1.39 (1.30,1.48) | <0.001 | 1.28 (1.21,1.36) | <0.001 |
|  |  |  |  |  |
| A competing risk analysis was implemented using the Fine-Gray subdistribution hazards model; death was regarded as a competing event | | | | |
|  |  |  |  |  |
| Subhazard ratios of greater than one indicate that higher numbers of systems medicated were related to greater incidence of the adverse event | | | | |
|  |  |  |  |  |
